# Computer-guided buccal cortical plate separation for removal of calcified benign odontogenic tumors affecting the mandibular angle region

**DOI:** 10.1186/s40902-022-00354-6

**Published:** 2022-09-22

**Authors:** Mohammed Omara, Ayman Gouda, Sherif Ali

**Affiliations:** Oral and Maxillofacial Surgery Department, Faculty of Oral and Dental Medicine, Cairo University, Cairo, Egypt

**Keywords:** Computer-guided surgery, Intrabony calcific odontogenic benign tumor, Patient-specific surgical guides, Buccal corticotomy, Buccal cortical plate separation

## Abstract

**Purpose:**

Surgical removal of intra-bony calcific benign lesions is technically challenging regarding its accessibility, proximity to vital structures, and deteriorating effect on the remaining bony structures.

**Methods:**

Computer-guided buccal cortical plate separation was performed for ten patients using patient-specific osteotomy locating guides and pre-bent plates. The guide was designed to outline the osteotomy, the buccal cortical plate was separated, the lesion was removed, and finally, the pre-bent plates were used to fix the separated cortex.

**Results:**

Surgical procedures were uneventful for all patients, operation time was 39.5 ± 13.01 min, postoperative pain decreased within the follow-up time intervals, and there was a statistical significant difference between the time intervals (*P* value < 0.001). Edema and trismus were acceptable. One case showed nerve affection which resolved after 4 weeks.

**Conclusion:**

Computer-guided buccal cortical plate separation for removal of intra-bony calcified benign lesions provides a promising approach, especially for inexperienced surgeons.

**Trial registration:**

ClinicalTrials.gov NCT05329974. Registered on 6 April 2022—retrospectively registered.

## Introduction

Odontogenic tumors (OTs) have captured the attention of pathologists and surgeons despite their rarity, owing to their devastating impact on the patient’s quality of life [1, 2]. In an ongoing attempt to address the wide diversity of odontogenic tumors, its classification has passed through many stages, starting from the first WHO classification released in 1971 till the fourth and latest classification released in 2017 [3–5]. The classification reflects the heterogeneity of the odontogenic tumors with respect to their origin either epithelial and/or mesenchymal tissues. Moreover, OTs differ greatly in their nature and behavior varying from completely benign lesions to locally invasive benign lesions and even malignant lesions [4, 6]

OTs’ inconsistent clinical behavior was reflected in their treatment methods, as some lesions may require no treatment, and others may be treated by conservative enucleation and curettage while others may require a more radical treatment via resection with safety margins [7]. Regardless of the method of treatment, the success of OTs treatment depends primarily on its complete removal to avoid any possible recurrence [8–10]. However, this complete removal may be complicated by their size, consistency, nature, and location [11].

The mandibular angle region is the site of predilection for various odontogenic tumors among them benign calcified odontogenic tumors [12–17]. Despite their benign nature, the excision of such calcified lesions indicates wide surgical access and extensive bone removal. Intra-oral total or staged excision of the lesion via either the buccal or lingual cortex has been illustrated in the literature [18–24]. The buccal approach is associated with extensive bone removal in the external oblique ridge which highly compromises mandibular angle strength. While the lingual approach reduces mandibular fracture risk, it endangers the lingual nerve; furthermore, the approach is technically challenging with limited accessibility [25].

In 2005, a new approach was introduced for the removal of deeply impacted third mandibular molars using buccal cortical plate separation (buccal corticotomy). In this approach a rectangular buccal cortical plate window was made over the tooth and separated, then the tooth was removed, and finally, the separated bone was seated and fixed in position [26–28].

Over the last two decades, computer-assisted surgery has been widely used in oral and maxillofacial surgery and moved from virtual planning to the construction of different patient-specific hardware. These advances markedly facilitate the surgical procedures and reduce intraoperative time, especially with inexperienced surgeons [29, 30]. In this study, we aim to assess the use of a computer-guided buccal cortical plate separation approach using patient-specific cutting guide for the removal of intra-bony calcified odontogenic lesions affecting the mandibular angle region.

## Patients and methods

**Fig. 1:**
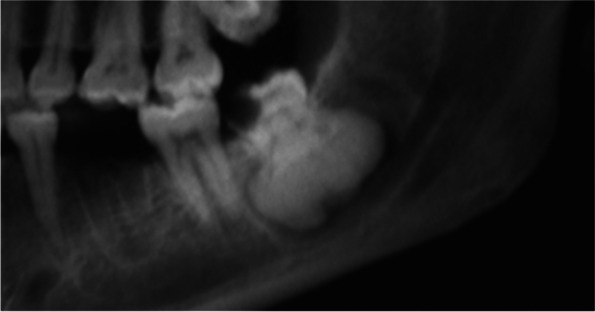
Preoperative radiograph

This was a prospective case series conducted on 10 patients with well-defined radiopaque lesions affecting the angle region, recruited consecutively from our out-patient clinic. The study was approved by the research ethics committee (IRB: 18,221) and followed the Declaration of Helsinki on medical research. Patients were selected according to the following clinical criteria: patients with well-defined radiopaque lesion affecting the angle region, its conventional removal may compromise the bone continuity and leads to pathological fracture indicating the need of buccal cortical plate separation technique. Patients with any medical condition contraindicating the surgical procedures were excluded (Fig. 1).

All enrolled patients were subjected to a computer-guided buccal cortical plate separation approach for removal of the calcific intra-bony masses using patient-specific osteotomy locating surgical guide and pre-bent titanium mini-plate either 2.0 for small lesions or 2.3 for large lesions compromising the inferior mandibular border based on the preoperative virtual planning and simulation.

### Preoperative preparation and virtual planning

**Fig. 2:**
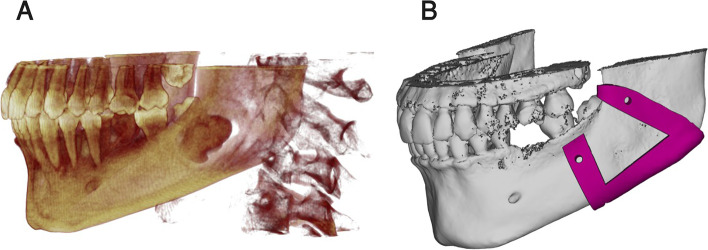
Preoperative virtual planning. **A** 3D image showing the extension of the lesion in the bone. **B** Patient-specific osteotomy locating guide constructed on the bone surface

Cone beam computed tomography (CBCT) (SCANORA 3D with Auto-Switch; Soredex, Helsinki, Finland) was requested for all patients. DICOM files were imported to the planning software (Mimics 19.0, Materialise NV, Leuven, Belgium). Intra-bony lesion size and extensions were further 3D analyzed after clinical assessment to assure their correspondence with eligibility criteria and prepare the surgical guide for the mass removal. The virtual planning was performed using the software to formulate the buccal window that totally exposes the intra-bony calcific mass without affection of neighboring vital structure, and the patient-specific osteotomy locating guide was then designed on the bone surface to outline the buccal window (Fig. 2).

**Fig. 3:**
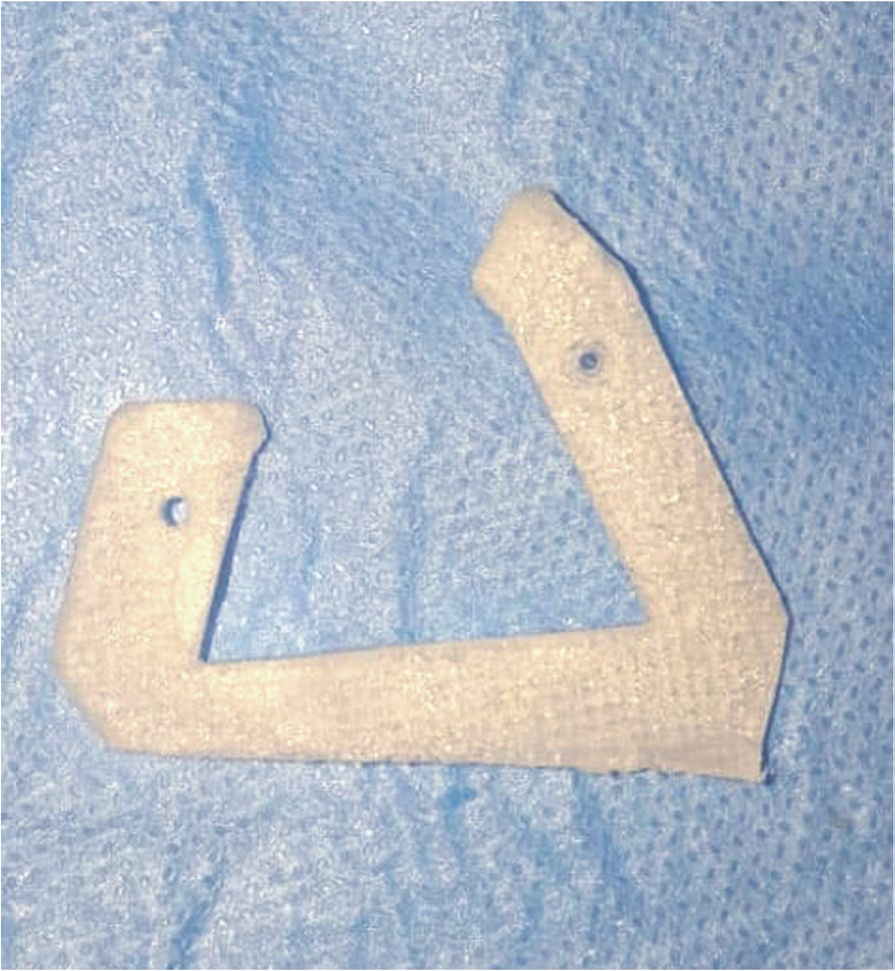
Printed patient-specific osteotomy locating guide

Finally, Stereolithography files (STL) of the surgical guide and the mandible were exported to an additive Computer-Aided Manufacturing (CAM) machine (FORMIGA P 110 printer; EOS e-manufacturing solutions, Munich, Germany) and manufactured in white polyamide (PA2200; EOS e-manufacturing solutions, Munich, Germany) using fused deposition modeling (FDM) technology (Fig. 3). The printed mandibular model was used for preoperative adaptation of one conventional either 2.0 or 2.3 titanium mini-plate based on the size of the lesion. The plate was adapted over the mandibular model to rest on sound bone mesial and distal to the created buccal window.

### Surgical procedures

**Fig. 4:**
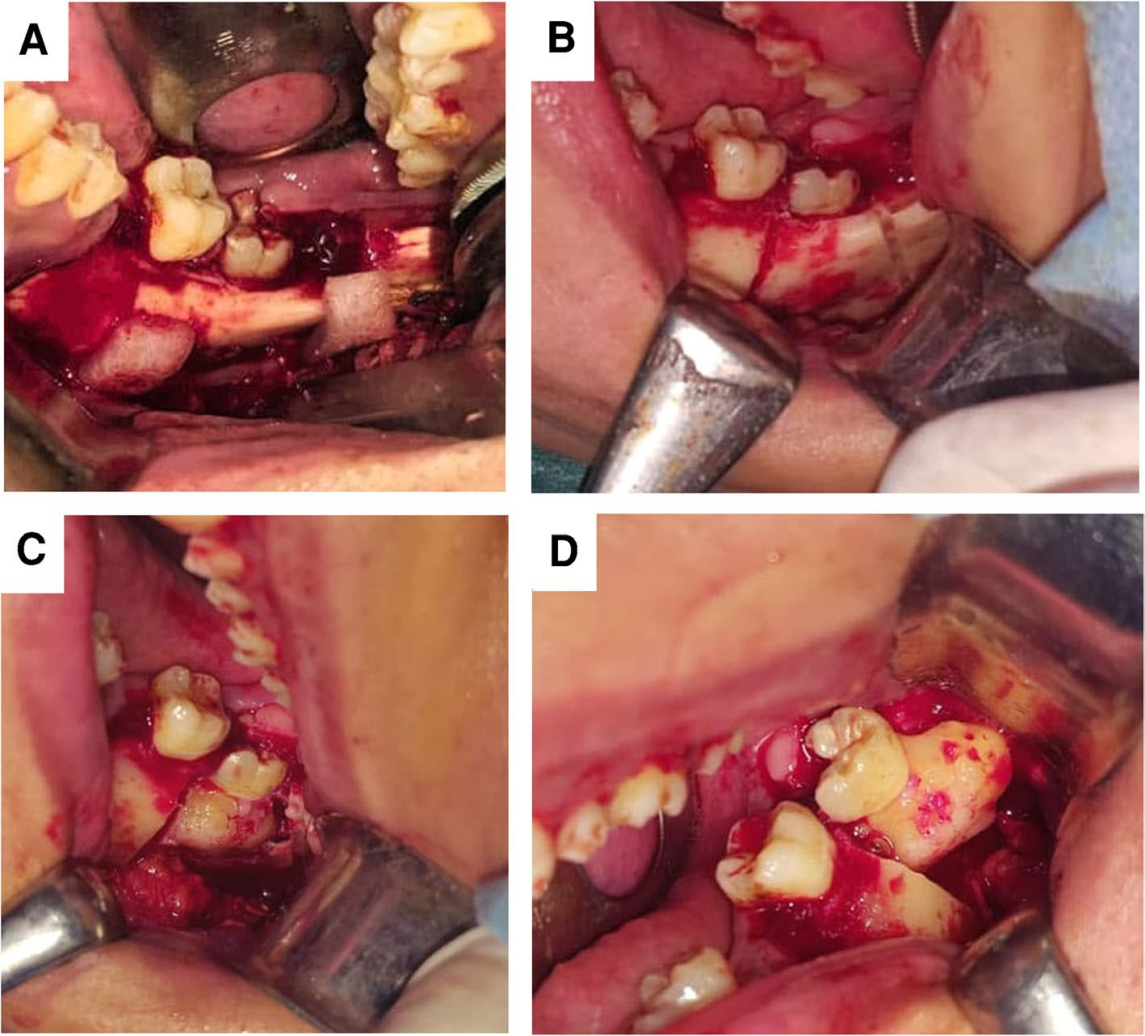
Surgical procedures. **A** The surgical guide fixed in place. **B** Osteotomy performed according to the preoperative plane. **C** Buccal cortical plate removed from position exposing the intra-bony calcific mass. **D** The exposed mass passively removed

**Fig. 5:**
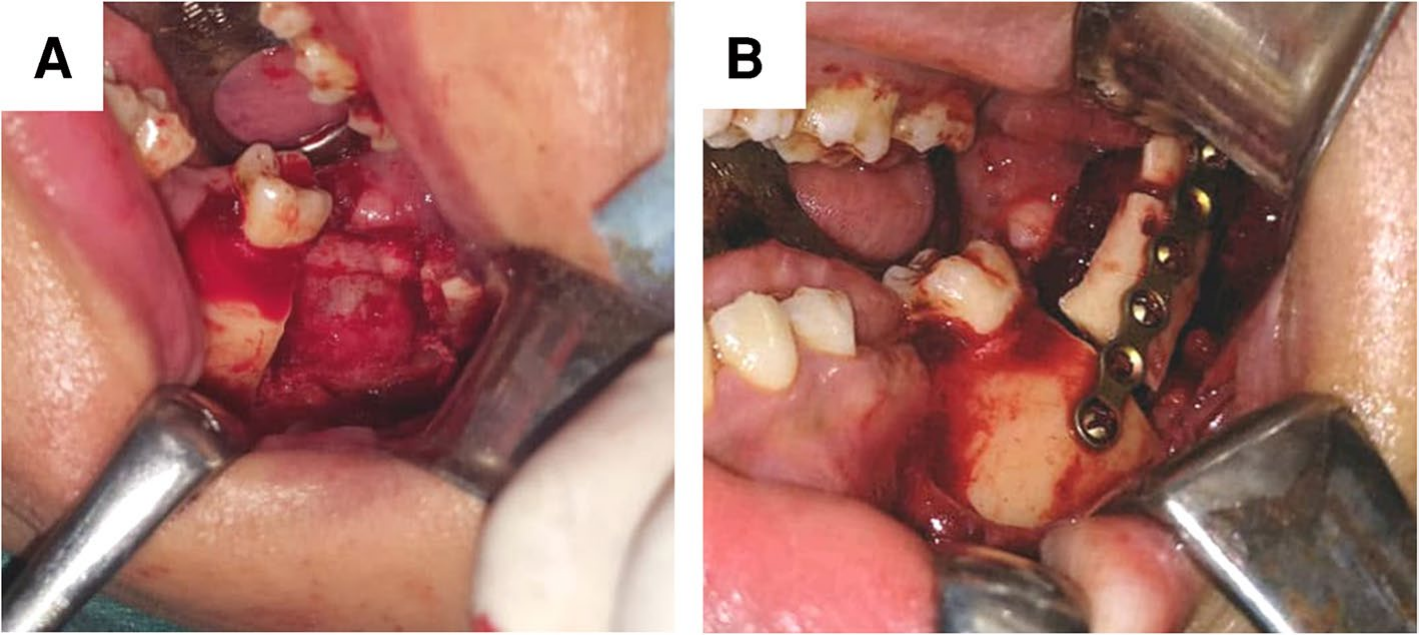
Surgical procedures. **A** The lesion bed after the calcified mass removal. **B** Buccal cortical plate placed and fixed in position

Surgical procedures were performed under general anesthesia with nasal intubation. Angel region was exposed through intraoral extended wards’ incision. The osteotomy locating surgical guide was placed on the exposed bony surface and fixed in position with 2 screws, then a reciprocating saw was used to perform the planned osteotomy. The created buccal door was mobilized using spatula chisels and opened to expose the intra-bony calcific mass. The exposed masses were passively removed either without or with sectioning according to the mass size (Fig. 4). The lesion bed was debrided, and the separated buccal cortical plate was repositioned and fixed using the pre-bent plate (Fig. 5). Finally, the wound was sutured.

### Postoperative follow-up and outcomes

A pressure band was applied to the cheek areas for 48 h postoperatively. The patients were instructed to apply ice packs over the cheek area for 20 min every hour for 6 h postoperatively and to rinse their mouth with warm saline solution starting on the second day after surgery. The patients were kept on a soft diet for the first 48 h. Postoperative antibiotic, analgesic, and anti-inflammatory drugs were prescribed for 5–7 days. The patients were recalled 2, 5, and 10 days after the surgery for initial clinical assessment.

Intra-operative time was measured from the start of the incision till suturing. Postoperative pain was evaluated 2, 5, and 10 days after the surgery using 0–10 visual analog scale (VAS). Edema and trismus were evaluated 5 and 10 days after the surgery. Edema was assessed using a four-grade scale: grade 0, no edema; grade 1, mild edema (just visible); grade 2; moderate edema (local); and grade 3, severe edema (extended). Trismus was also assessed using four grades scale: grade 0, no trismus; grade 1, mild trismus (the patient could insert two fingers vertically together into the mouth); grade 2; moderate trismus (the patient could insert only one finger); and grade 3, severe trismus (the patient could not open to insert one finger). Inferior alveolar nerve function was assessed at the follow-up time intervals by pricking using a dental probe at multiple points to evaluate the pain perception and by light touch using a cotton wisp to evaluate the tactile sensation. Further clinical assessment was continued at 4 weeks, 6 weeks, and 2 months to monitor the soft tissue healing and detect any plate exposure. Finally, a panoramic radiograph was requested after 2 months of surgery to assess the bone healing and integrity.

### Statistical analysis

Statistical analysis was performed using SPSS (Statistical package for the social sciences- IBM® SPSS® Statistics Version 20 for Windows, IBM Corp., Armonk, NY, USA). Qualitative data were represented as percentage and frequency. Quantitative data were represented as mean ± standard deviation. Kruskal–Wallis test was used to compare pain scores between the three-time points, and Bonferroni correction to post hoc multiple comparisons. The results were considered statistically significant if the *p* value was less than 0.05.

## Results

**Fig. 6:**
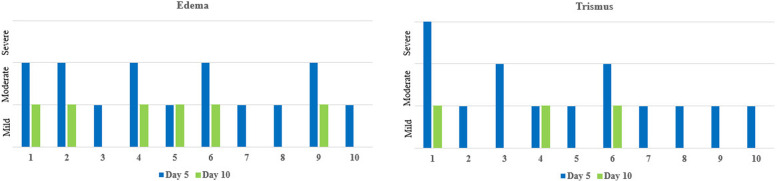
Bar charts showing edema and trismus in individual patients

**Fig. 7:**
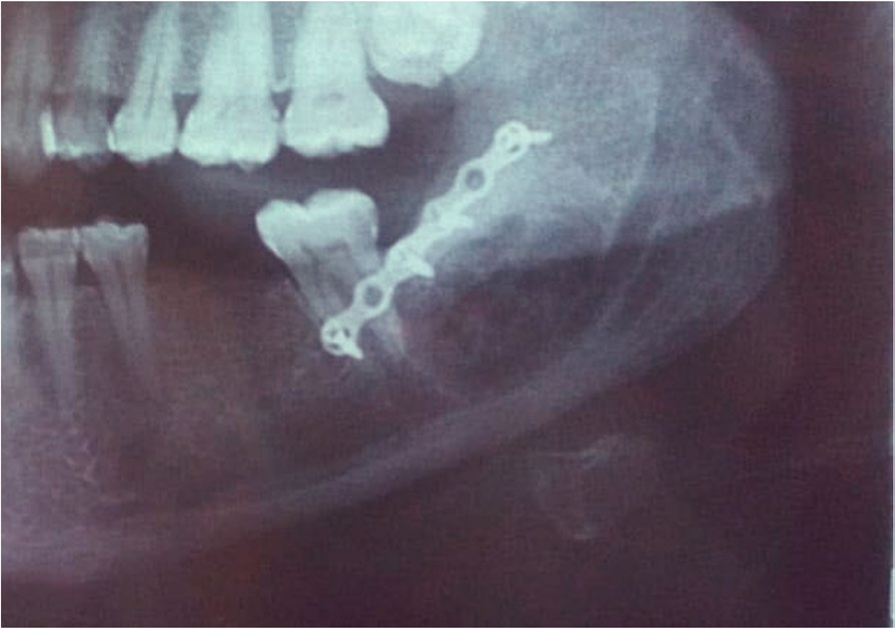
Postoperative radiographs after 2 months showing complete bone healing and integrity of the osteotomized buccal cortex

This study was conducted on 10 patients (5 males and 5 females) with well-defined radiopaque lesions affecting the angle region removed using a computer-guided buccal cortical plate separation technique. The mean age of the patients was 27.9 ± 3.7 years. Histological analysis showed that the lesion was complex odontoma in 6 patients (60%), Cementoblastoma in 4 patients (40%) (Table 1). The surgical procedures were uneventful for all patients, and the operation time was 39.5 ± 13.01 min. There was no nerve affection during the follow up time intervals, except only for one case who showed total nerve recovery 4 weeks postoperatively. The pain score decreased with time from 4.7 ± 1.49 at the 2nd day to 2.4 ± 1.07 at the 5th day, and 0.4 ± 0.52 at the 10th day, and there was a statistical significant difference between the 3 time points (*P* value < 0.001). Post hoc analysis showed no statistical significance difference between 2nd day and 5th day pain (*P* value 0.091); however, it showed a statistical significant difference between 5th day and 10th day pain (*P* value 0.026). At the 5th day, edema was moderate in 5 patients (50%) and mild in the other 5 patients (50%), while trismus was severe in one patient (10%), moderate in 2 patients (20%), and mild in 7 patients (70%). At the 10th day, no edema was observed in 4 patients (40%) and 6 patients (60%) showed mild edema, mild trismus was observed in 3 patients (30%), and no trismus in 7 patients (70%) (Fig. 6). Postoperative panoramic radiograph showed complete bone healing and integrity of the osteotomized buccal cortex (Fig. 7).

**Table 1:** Showing demographic data and medical information

Patient no	Age	Sex	Site	Size (mm)	Pathological diagnosis
1	35	Female	B/A	35 × 28 × 15	Complex odontoma
2	25	Female	B/A	28 × 14 × 7	Complex odontoma
3	24	Female	A	15 × 20 × 10	Cementoblastoma
4	32	Male	B/A	29 × 26 × 14	Complex odontoma
5	30	Male	A	15 × 15 × 12	Cementoblastoma
6	29	Female	B/A	34 × 25 × 14	Complex odontoma
7	26	Male	B/A	20 × 22 × 10	Cementoblastoma
8	25	Female	A	16 × 14 × 13	Cementoblastoma
9	24	Male	B/A	23 × 18 × 15	Complex odontoma
10	29	Male	B/A	21 × 20 × 15	Complex odontoma

*Abbreviations*: *B/A* Mandibular body angel region & *A* Mandibular angel region

## Discussion

Mandibular molar–angle region represents the most common site for odontogenic calcified benign tumors, that originate from the cellular remnants of the developing wisdom tooth that is usually associated with many molecular and physiological changes [12–17]. Different surgical approaches have been utilized for tumor removal at this site. Sagittal split osteotomy via an intra-oral approach avoids large bony defects without compromising the perfect surgical access, yet it may result in injury to the inferior alveolar nerve, possible condylar sag, and unpredicted cortical bone fracture [23, 24]. Intra-oral excision through the buccal or lingual cortex has been utilized as a simpler alternative [25]. However, these approaches are associated with high morbidity due to the excessive bone removal and nerve endangerment.

Buccal cortical plate separation (buccal corticotomy) has been introduced for deeply impacted third mandibular molars removal by Kwon Y-D. et al. [26] in a case report, followed by Tay ABG et al. [27] in another one. This approach is considered as an intermediate link between buccal cortex removal and sagittal split osteotomy. It avoids extensive bone removal, limited visibility, high risk of nerve injury, and mandibular fracture associated with the conventional buccal approach; moreover, it avoids the possible occlusion risk and unfavorable split associated with sagittal split osteotomy. Additionally, it is considered a straightforward and easier procedure compared to sagittal split osteotomy [26–28].

A previous case report by Nogueira et al. used free-hand buccal cortical plate separation for the removal of complex odontoma [31]. In this study, we used computer-guided buccal cortical plate separation using patient-specific cutting guide to overcome the pit flows of previous techniques for the removal of odontogenic calcified benign tumors affecting the molar angle region. The computer guidance revolution in dentistry allowed us to gain the advantage of the virtual planning and computer-assisted surgery to determine the extent of the bone window and outline it using the patient-specific cutting guide. Additionally, a printed 3D model was constructed for preoperative plate adaptation to facilitate the surgical procedures [30].

In our study, the patient’s age was mostly in the third decade which was coincident with the literature that most of the benign calcified odontogenic tumors start to appear accidentally during routine radiographic interpretation or after induction of slight swelling and discomfort at this age group [11, 32, 33]. Postoperative histological interpretation revealed complex odontomas in 6 patients. This is also coincident with the literature that demonstrated that odontomas represent the first and most common calcified odontogenic tumor and that posterior odontomas are mostly complex types [11, 34], while in the other 4 cases, the lesion was cementoblastoma, where posterior mandible represents the most common site of this lesion [35–37].

The use of a patient-specific surgical guide and pre-bent plate in our study facilitated the safe buccal cortex separation and accurate postoperative readaptation with low effort and minimal time. The wide access and visibility of the lesion facilitated the rapid removal of any undercut with safe excision of the calcified lesion without any induced forces this was reflected positively on the postoperative outcomes that revealed minimum pain, edema, and trismus with maximum inferior alveolar nerve integrity. These findings can be attributed to the wide approach associated with adequate access and maximum visibility of both lesion and vital structures with maximum preservation of the external oblique ridge integrity and mandibular strength [31]. However, the major limitation of this technique is very large lesions associated with thinning of the buccal cortex which contraindicate the buccal cortical plate separation [31].

## Conclusion

Surgical removal of intra-bony calcific benign lesions is technically challenging regarding its accessibility, proximity to vital structures, and deteriorating effect on the remaining bony structures. Computer-guided buccal cortical plate separation for intra-bony benign calcified odontogenic tumor removal provides a promising approach with minimal postoperative complications and facilitates surgical procedures, especially for inexperienced surgeons. However, we recommend the conduction of more investigations and comparative studies for further evaluation of its benefits compared to the conventional approach.

## Data Availability

All data are available whenever requested.

## Abbreviations

OTs: Odontogenic tumors
WHO: World Health Organization
CBCT: Cone beam computed tomography
STL: Stereolithography files
CAM: Computer-Aided Manufacturing
FDM: Fused deposition modeling
VAS: Visual analog scale
